# Area Deprivation and COVID-19 Incidence and Mortality in Bavaria, Germany: A Bayesian Geographical Analysis

**DOI:** 10.3389/fpubh.2022.927658

**Published:** 2022-07-15

**Authors:** Kirsi Marjaana Manz, Lars Schwettmann, Ulrich Mansmann, Werner Maier

**Affiliations:** ^1^Institute for Medical Information Processing, Biometry and Epidemiology (IBE), Ludwig-Maximilians-Universität (LMU), Munich, Germany; ^2^Helmholtz Zentrum München - German Research Center for Environmental Health (GmbH), Institute of Health Economics and Health Care Management, Neuherberg, Germany; ^3^Department of Economics, Martin Luther University Halle-Wittenberg, Halle, Germany; ^4^Pettenkofer School of Public Health, Ludwig-Maximilians-Universität (LMU), Munich, Germany

**Keywords:** COVID-19, area deprivation, standardized incidence ratio, standardized mortality ratio, Bavarian Index of Multiple Deprivation, hierarchical models

## Abstract

**Background:**

Area deprivation has been shown to be associated with various adverse health outcomes including communicable as well as non-communicable diseases. Our objective was to assess potential associations between area deprivation and COVID-19 standardized incidence and mortality ratios in Bavaria over a period of nearly 2 years. Bavaria is the federal state with the highest infection dynamics in Germany and demographically comparable to several other European countries.

**Methods:**

In this retrospective, observational ecological study, we estimated the strength of associations between area deprivation and standardized COVID-19 incidence and mortality ratios (SIR and SMR) in Bavaria, Germany. We used official SARS-CoV-2 reporting data aggregated in monthly periods between March 1, 2020 and December 31, 2021. Area deprivation was assessed using the quintiles of the 2015 version of the Bavarian Index of Multiple Deprivation (BIMD 2015) at district level, analyzing the overall index as well as its single domains.

**Results:**

Deprived districts showed higher SIR and SMR than less deprived districts. Aggregated over the whole period, the SIR increased by 1.04 (95% confidence interval (95% CI): 1.01 to 1.07, *p* = 0.002), and the SMR by 1.11 (95% CI: 1.07 to 1.16, *p* < 0.001) per BIMD quintile. This represents a maximum difference of 41% between districts in the most and least deprived quintiles in the SIR and 110% in the SMR. Looking at individual months revealed clear linear association between the BIMD quintiles and the SIR and SMR in the first, second and last quarter of 2021. In the summers of 2020 and 2021, infection activity was low.

**Conclusions:**

In more deprived areas in Bavaria, Germany, higher incidence and mortality ratios were observed during the COVID-19 pandemic with particularly strong associations during infection waves 3 and 4 in 2020/2021. Only high infection levels reveal the effect of risk factors and socioeconomic inequalities. There may be confounding between the highly deprived areas and border regions in the north and east of Bavaria, making the relationship between area deprivation and infection burden more complex. Vaccination appeared to balance incidence and mortality rates between the most and least deprived districts. Vaccination makes an important contribution to health equality.

## Introduction

COVID-19 (Coronavirus disease 2019), an infectious disease in humans caused by the Severe Acute Respiratory Syndrome Coronavirus-2 (SARS-CoV-2), first emerged in December 2019 in China and was declared a pandemic by March 2020 (1). This pandemic caused a significant increase in mortality and led to a heavy burden in healthcare systems worldwide. The search for factors related to COVID-19 outcomes is of great interest for the development of strategies to cope with this disease.

Lack of material and social resources may explain why worse health outcomes are often observed for residents of more deprived areas (2). Area deprivation relates to a large number of adverse health outcomes, e.g., coronary heart disease, type 2 diabetes or cancer (3–6). To analyze those area-level health disparities, deprivation indices are widely used (7–9). These indices include several distinct indicators or rather domains to describe different aspects of area-based lack of resources. Typically, income and employment are key domains of deprivation indices, but other indicators, e. g. educational or environmental aspects, are also considered.

Associations between aspects of COVID-19 and area deprivation have been investigated for several countries, including Germany (10–18). Even though COVID-19 is transmitted by individuals, these studies consistently report that the risk of COVID-19 infections, as well as COVID-19-related mortality, are higher in more deprived than in less deprived areas. However, it has also been found that this relationship could change over time (19, 20).

With regard to COVID-19 it should therefore be of interest to include data over longer time periods to account for temporal variations and to disentangle the associations between different deprivation domains and COVID-19 incidence and mortality.

The very first SARS-CoV-2 outbreak in Germany was reported for the southern federal state of Bavaria (21, 22), which is the largest German state by area and the second largest by population with more than 13 million inhabitants. This exceeds significantly the population size in many European countries. Moreover, Bavaria is one of the wealthiest states in an already economically strong country like Germany.

Infection control measures in Germany differ from state to state. Therefore, it would be appropriate to focus on a specific area with largely uniform control measures like Bavaria.

Several infection waves have occurred since March 2020 and at the time of submission of this paper, Bavaria and Germany are in the transition phase between the fifth and sixth infection wave. To date, Bavaria still lacks a comprehensive analysis on how area deprivation and the COVID-19 burden relate to each other over the course of the pandemic.

However, it should be considered that geographical patterns of health inequalities already existed before the pandemic. In particular, non-communicable diseases are unequally distributed in the population which could be related to area deprivation (3–6). Such geographical patterns of health inequality may also apply to COVID-19.

We therefore decided to focus on Bavaria to investigate which area-related material and social factors could be associated with COVID-19 infection risk and mortality, and how they interact with approaches chosen to counteract COVID-19 such as vaccination or lockdown. Given pre-existing geographical health patterns, we expected higher COVID-19 infection and mortality rates in more deprived districts. We hypothesized that income, employment and education may be related to COVID-19, as these factors appear to be strongly related to the ability to work from home, to reduce contacts and to reduce mobility. We explore how the effect of the measures taken may interact with area deprivation.

The specific aim of our study is to investigate the association between area deprivation and standardized SARS-CoV-2 incidence and mortality ratios (SIR, SMR) between spring 2020 and winter 2021 at the district level in Bavaria, Germany, and to assess whether area deprivation consistently explains the variability in local population-adjusted COVID-19 incidence and mortality at specific time points and over longer periods.

## Materials and Methods

### Data Source and Structure

The Bavarian Health and Food Safety Authority (Bayerisches Landesamt für Gesundheit und Lebensmittelsicherheit, LGL) is the competent authority in Bavaria for the report of SARS-CoV-2 data and publishes the aggregated data of all local Bavarian health offices (23). We processed the data provided by the LGL at the individual level for the years 2020 and 2021, starting on March 1, 2020, until December 31, 2021, to exclude sporadic infections before March 2020. This period included data from the first to the fourth wave of infection (24): the first wave started in calendar week 10 in 2020 (03/02/2020) in Germany and lasted until 05/17/2020, followed by sustained low rates in the summer period in 2020 between 05/18/2020 and 09/27/2020. The second wave started on 09/28/2020 and lasted until 02/28/2021. The third wave started on 03/01/2021 and went on until 06/13/2021, followed by sustained low rates in 2021 (between 06/14/2021 and 08/01/2021). The fourth wave started on 08/02/2021 and continued until 12/26/2021 (24).

In Germany and Bavaria, incident cases are persons with laboratory-confirmed evidence of SARS-CoV-2 (direct pathogen detection). COVID-19 deaths are defined as death cases related to this infection (25). In practice, it is often difficult to decide to what extent the SARS-CoV-2 infection directly contributed to the death. Both groups, people who died directly from the disease (“death due to COVID-19”) and patients with pre-existing conditions who were infected with SARS-CoV-2 and for whom the cause of death cannot be clearly determined (“death with COVID-19”), are reported as COVID-19-related deaths. We rely on data from the LGL without knowing in detail how the COVID-19-related deaths were identified as such by the health offices/doctors.

While the exact date of death was used for the analysis of mortality, the incidence was analyzed using the reporting dates of the infection, which do not have to coincide with the date of onset of symptoms or the date of the first positive test result. The Bavarian data do not include the date of the first positive test result. We did not perform nowcasting (26) to correct for the reporting delay. For a part of the infections, the date of symptom onset is given. In order to take into account the natural course of the infection, we additionally considered a 14-day delay between infections and deaths (27). For the main analysis, the data were aggregated in monthly periods. Accordingly, the first period of aggregated data (“March 2020”) includes data on infections between 1 March and 31 March 2020 and data on deaths between 15 March and 14 April 2020.

Premature mortality is an important indicator for public health and the effectiveness of health systems (28, 29). Deaths below the age of 65 are considered premature, occurring at an age well-below the average life expectancy and being in many cases preventable. A high percentage of premature deaths is an indication of increased health risks in the population.

### Area Deprivation

Area deprivation was assessed at district level (“Kreisebene”) using the Bavarian Index of Multiple Deprivation (BIMD) for the reference year 2015 (BIMD 2015) (30). Bavaria consists of 96 rural and urban administrative districts. Both the BIMD and the area deprivation measure for the whole of Germany, the German Index of Multiple Deprivation (GIMD), were constructed based on the method used in the UK and adapted to the German context (6). Both indices have been shown to be associated with a number of adverse health outcomes in non-communicable diseases, including diabetes and cancer incidence, but also mortality (31–33).

The BIMD consists of seven deprivation domains with different weighting: income (25%, financial poverty of residents), employment (25%, unemployment), education (15%, lack of vocational training), municipal/district revenue (15%, financial situation of the districts), social capital (10%, for lack of social capital), environment (5%, poor quality of the physical environment), and security (5%, accidents and crime rates) (29, 30). In our analysis, we used quintiles of the overall score and the domain-specific scores (“deprivation quintiles”) where quintile 1 (Q1) includes 20% of the least deprived and quintile 5 (Q5) 20% of the most deprived districts. The BIMD quintiles were calculated using the distribution of districts without taking population size into account. We label the quintiles as follows: least deprived (Q1), less deprived (Q2), moderately deprived (Q3), more deprived (Q4), and most deprived (Q5). During the observation period, we assigned to each district a constant deprivation quintile not changing over time.

### Statistical Analyses

We determined SIR and SMR as ratios of observed to expected infection incidence and mortality rates. SIR and SMR are therefore relative risks. We calculated the expected values using indirect standardization to the latest available Bavarian population (from 2020) (34). The Bavarian population is comparable to the European Standard Population 2013 (see Supplementary Table 1). For this approach, the population was stratified into 15 age and sex-specific categories (0–4, 5–9,..., 60–64, 65–74, and 75 years and older). These age categories were available for each district. For each month, the stratum-specific event rates (infection/mortality) for the whole of Bavaria were multiplied by the specific population of the district. Summing over all strata gives the local expected number of events. The locally observed number of events was divided by the locally expected number to obtain the local standardized event ratio. These ratios can therefore change every month. Monthly, they represent the extent of heterogeneity of SIR and SMR in the Bavarian population.

The primary analysis examined the association between area deprivation and SIR and SMR simultaneously using the bivariate version of the Besag-York-Mollie (BYM) model (35–37). This model is referred to as a standard model in geographical epidemiology (38). The BYM model includes both rates as a bivariate endpoint and considers the correlation between them. It is important to include both measures together in the model (i) to increase power in detecting specific associations (39) and because (ii) the strength of correlation between the two measures is a measure of dependence on common, area-level, unmeasured risk factors (40). Another problem in working with spatial data is spatial autocorrelation, which occurs when the values of a variable (e.g., SIR) measured at nearby locations are more similar than the values of the same variable measured at a greater distance (41). The BYM model also takes into account that the relative risks (SIR, SMR) of neighboring districts are correlated and introduces smoothing of extremely large estimates, which are generally caused by few observations in small regional populations. As is often the case, only nearest neighbors (i.e., districts with a common border) are considered for the correlation in the model, while next nearest neighbors are not. Border effects are neglected, i.e., morbidity and mortality in neighboring districts outside Bavaria are not considered. Neighboring German federal states to Bavaria are Baden-Wuerttemberg and Hesse in the west, and Thuringia and Saxony in the north. Neighboring countries are the Czech Republic in the east, Austria in the east and the south, and Switzerland in the south of Bavaria.

The deprivation quintiles were included in the BYM model as an ordinal variable. In a test for linear trend, linear contrast was used for the quintiles centered on their mean (−2, −1, 0, 1, 2). In a sensitivity analysis, the BYM model for SIR/SMR using the BIMD was additionally adjusted for the population density for 2020 (“population density in 1,000 inhabitants per square kilometer”). In a complementary analysis, we also examined how area deprivation affects the risk of dying as an infected person using the standardized case fatality ratio (sCFR). The sCFR describes the ratio of the regional variation in mortality to the regional variation in the documented infection process (42). It estimates the relative risk of dying from or with COVID-19 as a documented case. Strong small-scale variability in sCFR suggests a preference for regional over higher-level measures to manage the incidence of infection. The sCFR can be estimated as the ratio of SMR and SIR using the bivariate BYM model (42).

The BYM model uses non-informative a priori distributions according to the default settings of the analysis software (40). We reported the mean estimates averaging over 20,000 replications, including an initial burn-in period of 10,000. Point estimates were reported together with 95% credibility intervals (95% CrI). Statistical significance is claimed if the 95% CrI does not contain the value one. These models are referred to as structured models.

In addition to the Bayesian BYM model, we fitted a frequentist multilevel Poisson model to the data. To determine a marginal effect of the BIMD 2015 on the SIR and the SMR over the entire observation period, multilevel Poisson models were used in which the district and time were random effects and the BIMD or one of the seven domains were fixed effects. These models are fitted using the maximum likelihood method and referred to as unstructured models in this paper. More specifically, these generalized mixed Poisson models include the observed counts as the outcome and the BIMD quintiles as the predictor (fixed effect). The logarithm of the expected counts was included as an offset and time and district as random effects. The observed and expected counts were aggregated beforehand on a monthly basis, using the same data as for the BYM model. The model provides risk ratios (RR) interpreted for BIMD as relative increase in SIR/SMR per one quintile increase in BIMD. In addition, population density was included as a fixed effect for the sensitivity analysis.

For testing statistical significance in the multilevel models, we adopted a hierarchical approach: first, the main effect of the association between BIMD and SIR/SMR was estimated and statistical significance was claimed at a 5% confidence level. If the main effect was found to be statistically significant, analyses were conducted for the seven domains. The *p*-values for the domains were adjusted using the Bonferroni correction. This hierarchical testing procedure maintains the overall significance level of 5% and minimizes the number of falsely significant results due to multiple testing for the same data. Point estimates are reported together with 95% confidence intervals (95% CI).

Additional exploratory analyses were conducted to examine the relationship between incidence rates (IR) and mortality rates (MR) between the most and least deprived districts (IR_Q5_/IR_Q1_ and MR_Q5_/MR_Q1_) over time.

The analyses were carried out using the software R (Version 3.6.3.) (43) and GeoBUGS (Version 1.2) (40). The unstructured models were estimated using the R function “glmer” of the R package “lme4” (44). The R function “supsmu” was used to smooth weekly incidence and mortality rates to present smoother curves (45). Maps were generated in QGIS 3.10.10 (46).

## Results

### Overview

**Figure 1:**
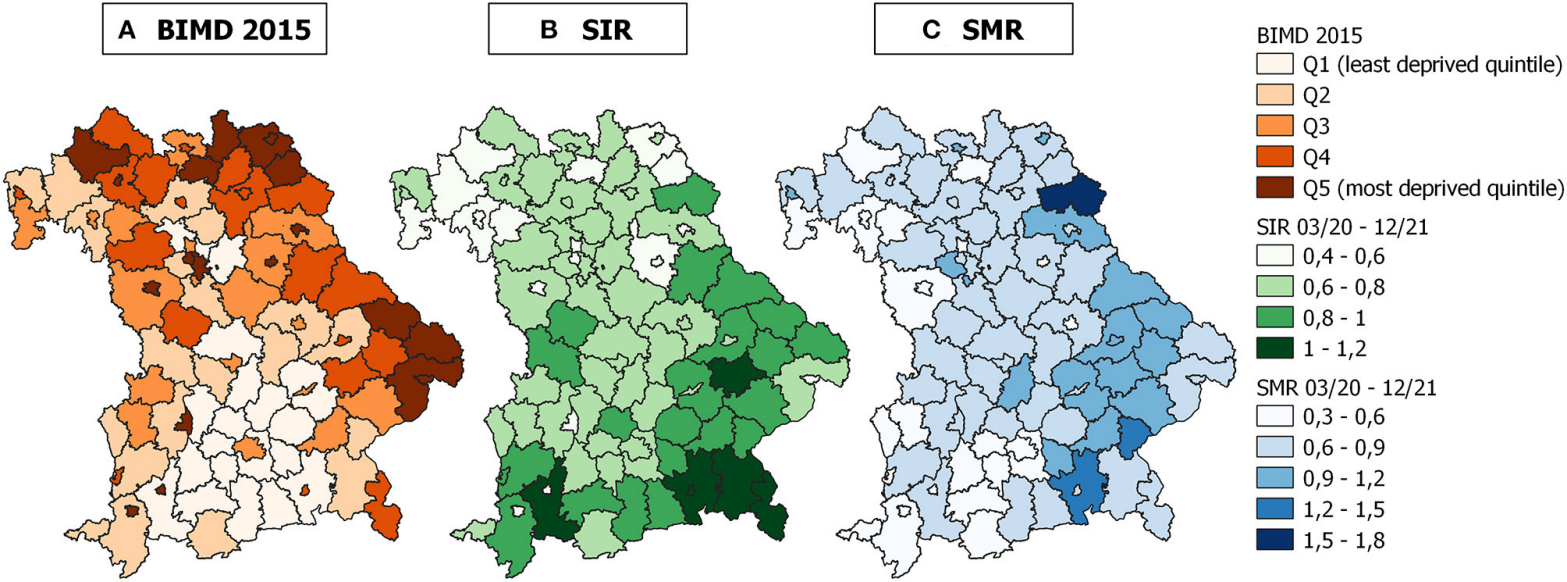
Maps of area deprivation and standardized incidence and mortality ratios in Bavaria, Germany. **(A)** Bavarian Index of Multiple Deprivation for the reference year 2015 (BIMD 2015), **(B)** standardized incidence ratio (SIR), and **(C)** standardized mortality ratio (SMR) for the 96 districts in Bavaria, Germany. SIR and SMR were calculated for the period between March 1, 2020 and December 31, 2021.

After excluding all individuals without valid age and/or sex information (n=10,993, 0.8% of data), a total of 1,319,456 SARS-CoV-2 infections and 19,571 associated deaths were reported in Bavaria between March 1, 2020 and December 31, 2021. Comparison of daily SARS-CoV-2 incidences and deaths in Bavaria compared to Germany and other selected European countries of similar size to Bavaria are shown in Supplementary Figures 1, 2, respectively. In persons under 65 years of age, a total of 1,694 COVID-19-associated deaths were reported (8.5% of all deaths). Infections were evenly distributed between both sexes (males 49.8%), and the median age of those infected was 38 years (interquartile range IQR: 22 to 54 years). Of the fatal COVID-19 cases, 52.2% were males with a median age of 83 years (IQR: 76 to 89 years). The population of the districts varies between 40,842 and 1,488,202 inhabitants, with a median of 117,648 inhabitants. Taking into account the area of the districts, the population density ranges from 66 to 4790 persons per square kilometer (median: 149). Of the 96 districts, 19 (20, 18, 20, and 19) belong to the first (second, third, fourth, and fifth) BIMD 2015 quintile, respectively (Figure 1A). As can be seen from Supplementary Figure 3, the assignment of districts to BIMD 2015 quintiles looks different for each domain of the BIMD 2015. In addition, Figures 1B,C show maps of SIR and SMR of accumulated data over the whole observation period.

### Unstructured Analysis of Time-Specific Area Deprivation Effects

**Figure 2:**
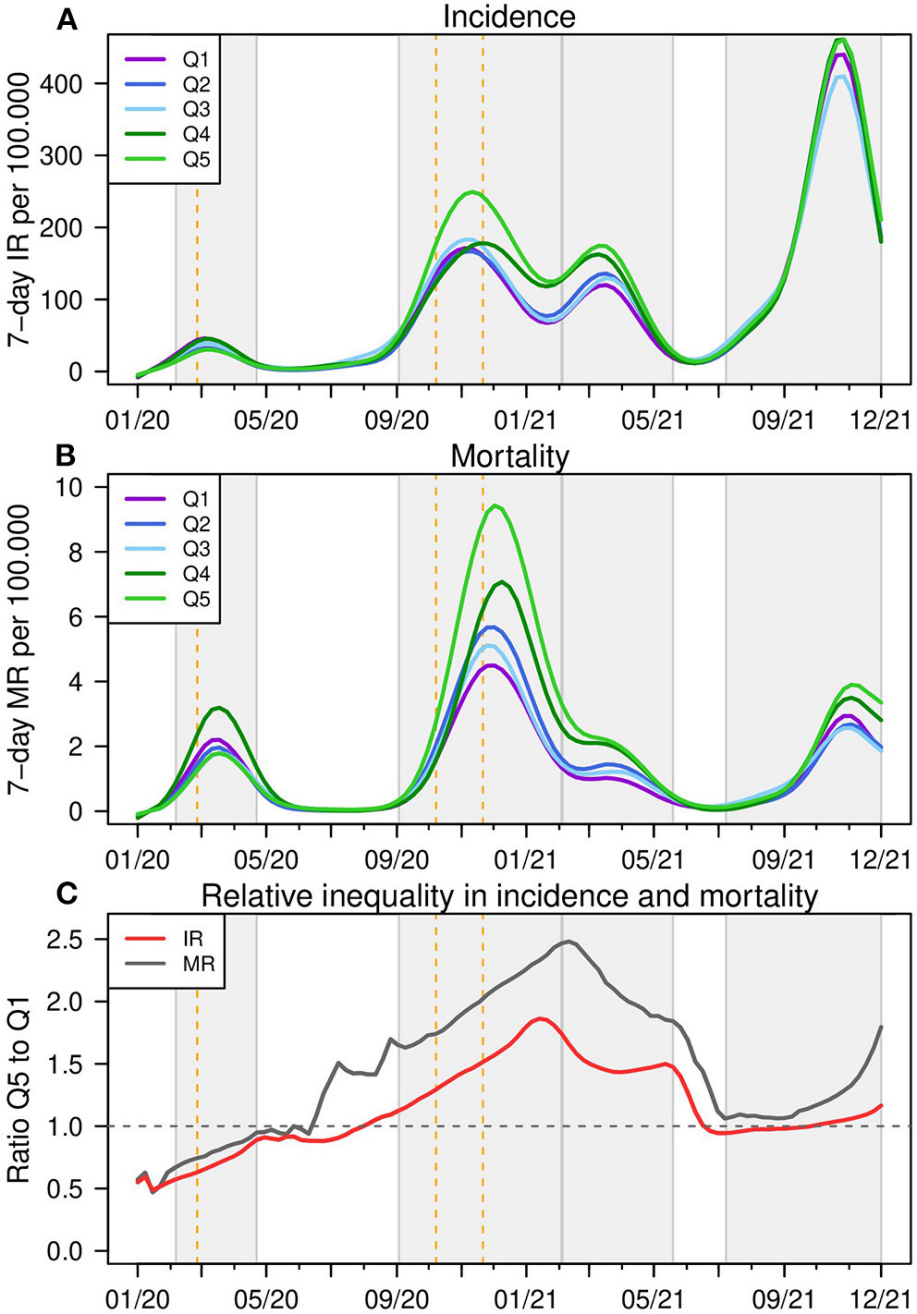
Incidence and mortality rates for BIMD 2015 quintiles in Bavaria, Germany. Weekly reported incidence **(A)** and overall mortality rates **(B)** per 100,000 and the ratio of Q5 and Q1 in **(C)** for districts belonging to each quintile of the Bavarian Index of Multiple Deprivation 2015 (BIMD 2015) between 2015 between January 2020 and December 2021 in Bavaria, Germany. Q1 describes the 20% least deprived and Q5 the 20% most deprived of all 96 districts. The time periods of the four infection waves are shown as light gray areas in the figure. IR, incidence rate, MR, mortality rate.

Figure 2 shows the weekly reported incidence and SARS-CoV-2-associated mortality rates in the districts belonging to each BIMD 2015 quintile. To indicate the first wave, the graph starts in January 2020 and covers the whole period until December 2021. The incidence rate (IR) curve (Figure 2A) thus qualitatively shows the four pandemic waves in Bavaria. The periods of the waves according to the official definition (23) are shown as light gray-shaded areas in Figure 2 and the initial dates of the lockdowns are indicated as vertical dashed lines.

During the first wave in March/April 2020, the least and more deprived districts (Q1 -purple and Q4 -green) have the highest IR, while the most deprived districts have the lowest rates (Q5 -light green). At the beginning of the second wave in August 2020, moderately deprived districts (Q3 -light blue) show the highest IR, but as the wave progresses, the most deprived districts are the most affected, peaking in October and November 2020. Around Christmas 2020, the districts in Q4 and Q5 show an increase in IR, and these two categories remain the ones with the highest IR until the end of the third wave. At the beginning of the fourth wave in August 2021, districts in Q1 and Q3 show higher rates. After the rapid increase in pandemic activity in September and October 2021, again the districts in Q4 and Q5 show the highest IR.

In terms of mortality rates (MR, Figure 2B), MR generally peak a few weeks after the peak in IR. During the first wave, MR are highest in the districts Q4 and Q1, similar to IR. The second MR peak is observed around Christmas 2020, with MR highest in Q4 and Q5 districts, which is also true for the third and fourth pandemic waves. Up to 2.5-fold relative differences between mortality rates are observed between Q5 and Q1. Premature mortality rates show a similar ranking of districts as mortality rates (see Supplementary Figure 4). However, because of the smaller sample size, the curves are not as smooth. In the second and in the third wave, the least deprived districts in Q1 show a nearly constant and very low MR. It is interesting to note that while overall COVID-19 mortality rates decline after the second wave, the magnitude of premature mortality remains about the same.

Figure 2C shows a remarkable ratio for IR and MR between the districts with the highest and lowest deprivation for the first COVID-19 winter. In the period of general vaccination (the first months in 2021), this inequality gets balanced.

### Unstructured Analysis of Overall Area Deprivation Effects

**Figure 3:**
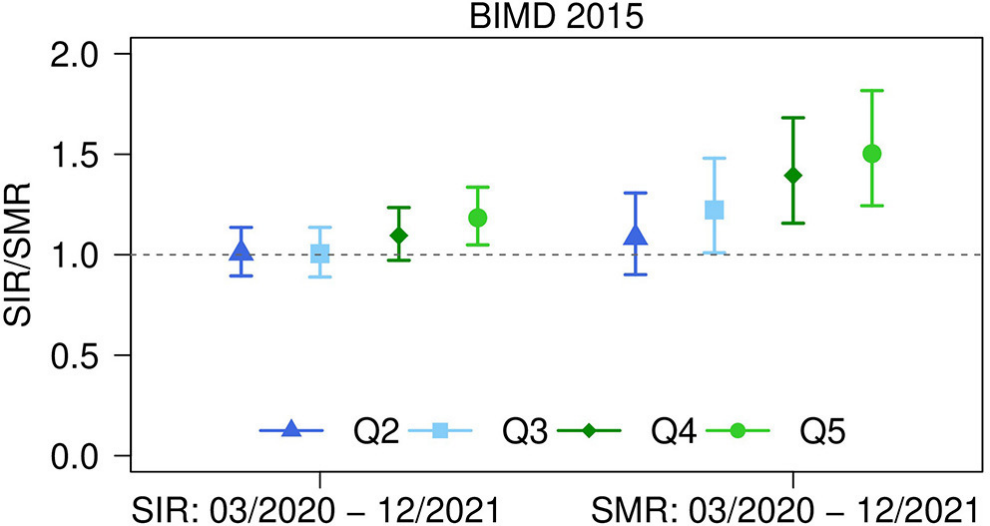
Strength of associations between area deprivation and standardized incidence and mortality ratios in Bavaria, Germany. Strength of associations between Bavarian Index of Multiple Deprivation (BIMD) 2015 and standardized incidence ratio (SIR) and standardized mortality ratio (SMR) for the 96 districts in Bavaria, Germany. Estimates for the period between March 1, 2020 and December 31, 2021 are shown for BIMD 2015 quintiles Q2 to Q5 (with Q1 = least deprived as the reference category) together with 95% confidence intervals. The estimates are adjusted for district and time.

The overall strength of the association between area deprivation, as measured by either the BIMD 2015 or its domains, and the SIR/SMR was calculated for the entire study period, adjusting for district and time. The results are shown in Figure 3 for the BIMD 2015 and in Table 1 for the BIMD 2015 and its seven domains. For the BIMD 2015, a statistically significant positive association was found with SIR and SMR (SIR = 1.04 (95% CI: 1.01 to 1.07), *p* = 0.002; SMR = 1.11 (95% CI: 1.07 to 1.16), p <0.001, per one quintile increase in the BIMD 2015). The SIR/SMR thus increases with increasing area deprivation.

**Table 1:** Strength of associations between area deprivation and standardized incidence and mortality ratios in Bavaria, Germany.

	**Model without population density**				**Model with population density**			
**Area deprivation index / domain**	**SIR (95% CI)**	***p***	**SMR (95% CI)**	***p***	**SIR (95% CI)**	***p***	**SMR (95% CI)**	***p***
BIMD 2015	**1.04 (1.01, 1.07)**	**0.002**	**1.11 (1.07, 1.16)**	**<0.001**	**1.05 (1.02, 1.08)**	**<0.001**	**1.12 (1.07, 1.17)**	**<0.001**
Income	**1.05 (1.02, 1.08)**	**0.003**	**1.11 (1.06, 1.16)**	**<0.001**	**1.05 (1.02, 1.08)**	**0.003**	**1.11 (1.06, 1.16)**	**<0.001**
Employment	1.02 (1.00, 1.05)	0.653	**1.09 (1.05, 1.14)**	**<0.001**	1.04 (1.01, 1.08)	0.105	**1.12 (1.07, 1.18)**	**<0.001**
Education	1.03 (1.00, 1.06)	0.155	1.06 (1.02, 1.11)	0.056	**1.04 (1.01, 1.07)**	**0.043**	1.07 (1.02, 1.12)	0.051
Municipal/district revenue	1.01 (0.99, 1.04)	1	1.05 (1.00, 1.10)	0.402	1.01 (0.98, 1.05)	1	1.07 (1.01, 1.13)	0.091
Social capital	**1.04 (1.02, 1.07)**	**0.010**	**1.11 (1.06, 1.16)**	**<0.001**	**1.04 (1.02, 1.07)**	**0.009**	**1.11 (1.06, 1.16)**	**<0.001**
Environment	**0.95 (0.93, 0.98)**	**0.005**	1.00 (0.95, 1.04)	1	**0.93 (0.90, 0.97)**	**<0.001**	0.98 (0.92, 1.04)	1
Security	1.02 (1.00, 1.05)	0.693	0.99 (0.95, 1.04)	1	1.03 (1.00, 1.06)	0.306	0.99 (0.94, 1.04)	1

*SIR, standardized incidence ratio; CI, confidence interval; SMR, standardized mortality ratio. Shown are models without (left) and with adjustment for population density (right). Ratios were calculated accumulating the data between 01/03/2020 and 12/31/2021 and adjusted for district and time. Statistically significant results are printed in bold. P-values for the Bavarian Index of Multiple Deprivation 2015 (BIMD 2015) domains are Bonferroni adjusted for multiple testing. A p-value after adjustment of >1 is coded as 1*.

With respect to the single domains, statistically significant positive associations were found for SIR and SMR with income deprivation (SIR = 1.05 (95% CI: 1.02 to 1.08), *p* = 0.003; SMR = 1.11 (95% CI: 1. 06 to 1.16), *p* < 0.001 per one quintile increase) and social capital deprivation (SIR = 1.04 (95% CI: 1.02 to 1.07), *p* = 0.010; SMR = 1.11 (95% CI: 1.06 to 1.16), *p* < 0.001 per one quintile increase). Another positive association was found for SMR with employment deprivation (SMR = 1.09 (95% CI: 1.05 to 1.14), *p* < 0.001 per one quintile increase) and a negative association for SIR with environmental deprivation (SIR = 0.95 (95% CI: 0.93 to 0.98), *p* = 0.005).

Table 1 also shows the estimates from a model adjusted for population density. The results are very similar to the results of the models without adjustment for population density, except for educational deprivation, which now shows a positive association with SIR.

### Structured Analysis of Time-Specific Area Deprivation Effects

**Figure 4:**
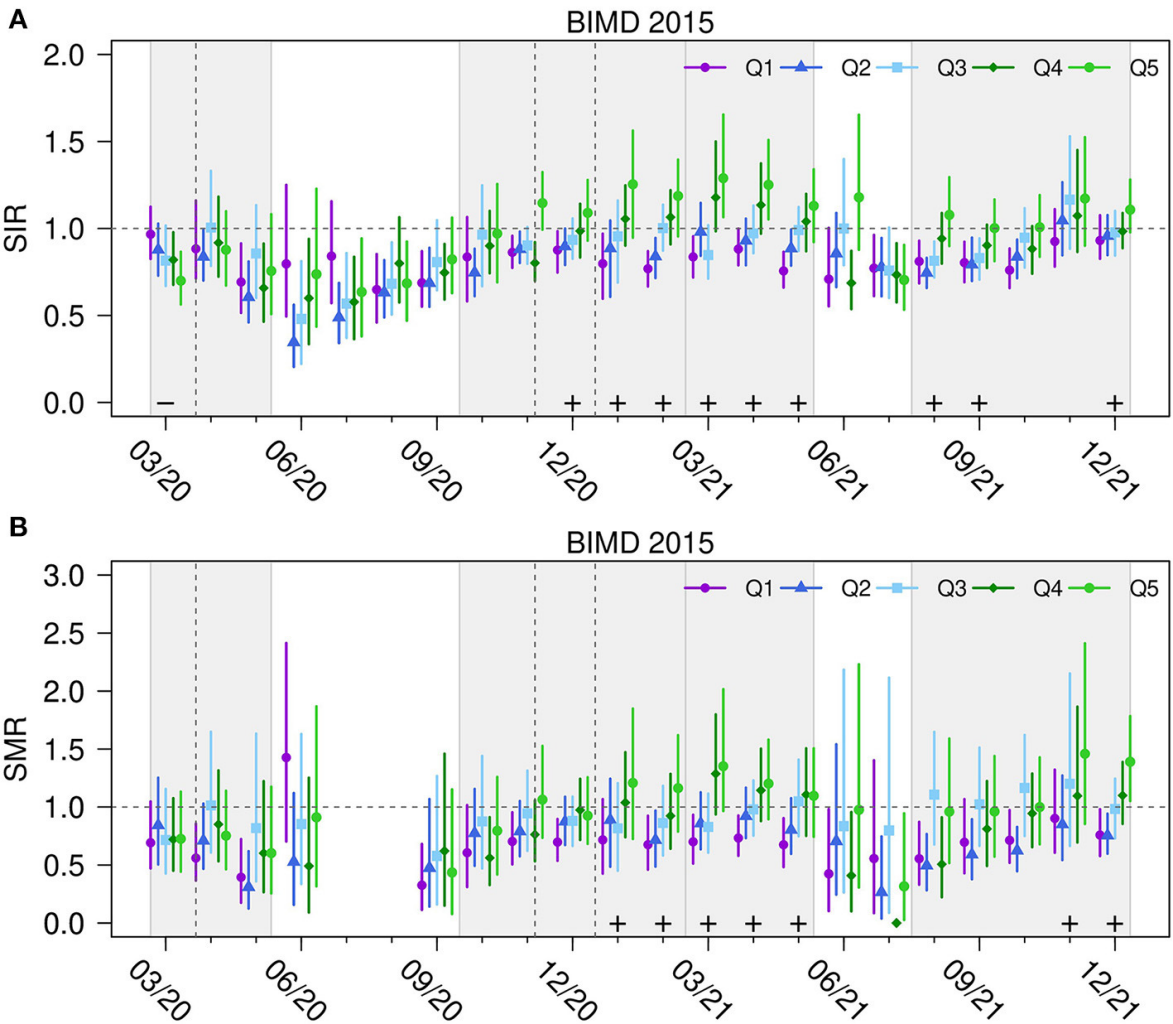
Standardized incidence and mortality ratios for BIMD 2015 quintiles in Bavaria, Germany. Standardized incidence ratios SIR **(A)** and mortality ratios SMR **(B)** of SARS-CoV-2 infections and related fatalities for quintiles Q1 (the 20% least deprived districts) to Q5 (the 20% most deprived districts) of Bavarian Index of Multiple Deprivation 2015 (BIMD 2015) between March 2020 and December 2021 in Bavaria, Germany. A plus sign (+) indicates a statistically significant increasing linear trend with increasing deprivation quantile. The time periods of the four infection waves are shown as light gray areas in the figure. The horizontal dashed gray line shows the value of one (“neither increased nor decreased SIR/SMR”). Vertical gray lines show the beginning of the lockdowns.

Figure 4 shows the estimates of the BYM model for the bivariate endpoint SIR (A) and SMR (B) over time for each quintile of the BIMD 2015. The point estimate for each quintile is shown along with the 95% CrI, and the value of one (“neither increased nor decreased SIR/SMR“) is shown as a dashed line. Note that due to the very low number of deaths in July and August 2020 in Bavaria (*n* = 11 and *n* = 18, respectively), the SMR could not be reliably estimated and is not shown in the figures.

At the beginning of the first wave in March 2020, the SIR are the highest in the least deprived districts and the lowest in the most deprived districts. However, as the first wave progresses, this effect disappears. Between the first and second wave in summer 2020, infection and death counts were low, which is reflected in wide credibility intervals. At the beginning of the second wave, districts from the two least deprived quintiles (Q1 and Q2) appear to have a slightly lower SIR. However, given the uncertainty of the estimates, no clear conclusion can be drawn from this. In December 2020, in the middle of the second wave, the trend of an increasing SIR with increasing area deprivation becomes statistically significant and remains so until the end of the third wave. The fourth wave begins with the same significant trend of higher incidence ratios in more deprived districts, and at the end of the fourth wave the effect is still present.

Mortality ratios show similar trends compared to incidence rates. In the last month (December 2021), the SIR was 0.93 (95% CrI: 0.83 to 1.07) for the least deprived and 1.11 (95% CrI: 0.98 to 1.28) for the most deprived districts. The corresponding numbers for SMR are 0.76 (95% CrI: 0.58 to 0.98) and 1.39 (95% CrI: 1.05 to 1.78). Figure 4 also implies that the association between the BIMD 2015 and SIR/SMR is strongly fluctuating over time (tests on time x BIMD interaction are highly significant with *p* < 0.00001).

In an additional sensitivity analysis we, included population density in the BYM model. The adjusted result is shown in Supplementary Figure 5. The results show the effect of the BIMD 2015 independent of population density. The overall impression is that the results remain comparable to the unadjusted model shown in Figure 4. Population density is generally higher in city districts than in rural districts. Therefore, the population-adjusted model partially controls for the effects of densely populated cities. In both the unadjusted and population-adjusted models, the linear trend of BIMD remains similar. Population density itself showed a statistically significant positive effect on the SIR mainly in between infection waves. This means that in times of low infection activity (summer 2020 and summer 2021), the SIR was higher in more densely populated districts.

Corresponding analyses for the seven domains of the BIMD 2015 are shown in Additional files 6 to 12. During the first wave, no clear association between income deprivation and SIR/SMR can be observed (Supplementary Figure 6). In the second and third waves, higher SIR and SMR are detected in districts with higher income deprivation. At the end of the fourth wave, this effect is also present for both SIR and SMR.

In the second and third wave, the SIR showed higher values in districts with higher employment deprivation (Supplementary Figure 7), which occasionally also applied to the SMR in winter 2020/2021. Between these waves, the association did not show any clear direction.

Educational deprivation (Supplementary Figure 8) showed a significant positive linear trend with SIR and SMR that started in the second wave and continued until the fourth wave. It appears that the association was significant either at the beginning and/or at the end of the waves.

For the time periods between the waves, there was a negative linear trend between municipal/district revenue deprivation and SIR (Supplementary Figure 9), implying that infection ratios are higher in districts with lower municipal/district revenue deprivation. During the waves, SIR was occasionally both positively and negatively associated with SIR and SMR.

Social capital deprivation (Supplementary Figure 10) was positively associated with SIR at the end of the second wave, and with SIR and SMR in the third wave and the second half of the fourth wave.

Environmental deprivation (Supplementary Figure 11) shows a positive and significant linear trend with SIR, mostly between waves where only small numbers of cases occur. This positive trend changed to a negative trend in the fourth wave, which is also true for the SMR.

Security deprivation (Supplementary Figure 12) shows hardly any relevant association with SIR/SMR.

The median correlation (over all time periods) between SIR and SMR in the models with the BIMD 2015 or the domains ranged from 0.74 (for the model with income deprivation, see Supplementary Figure 6) and 0.78 (for the models with employment and environmental deprivation, see Supplementary Figures 7, 11, respectively). This strong correlation between both endpoints suggests that both SIR and SMR have similar geographical risk patterns.

### Standardized Case Fatality Ratio

**Figure 5:**
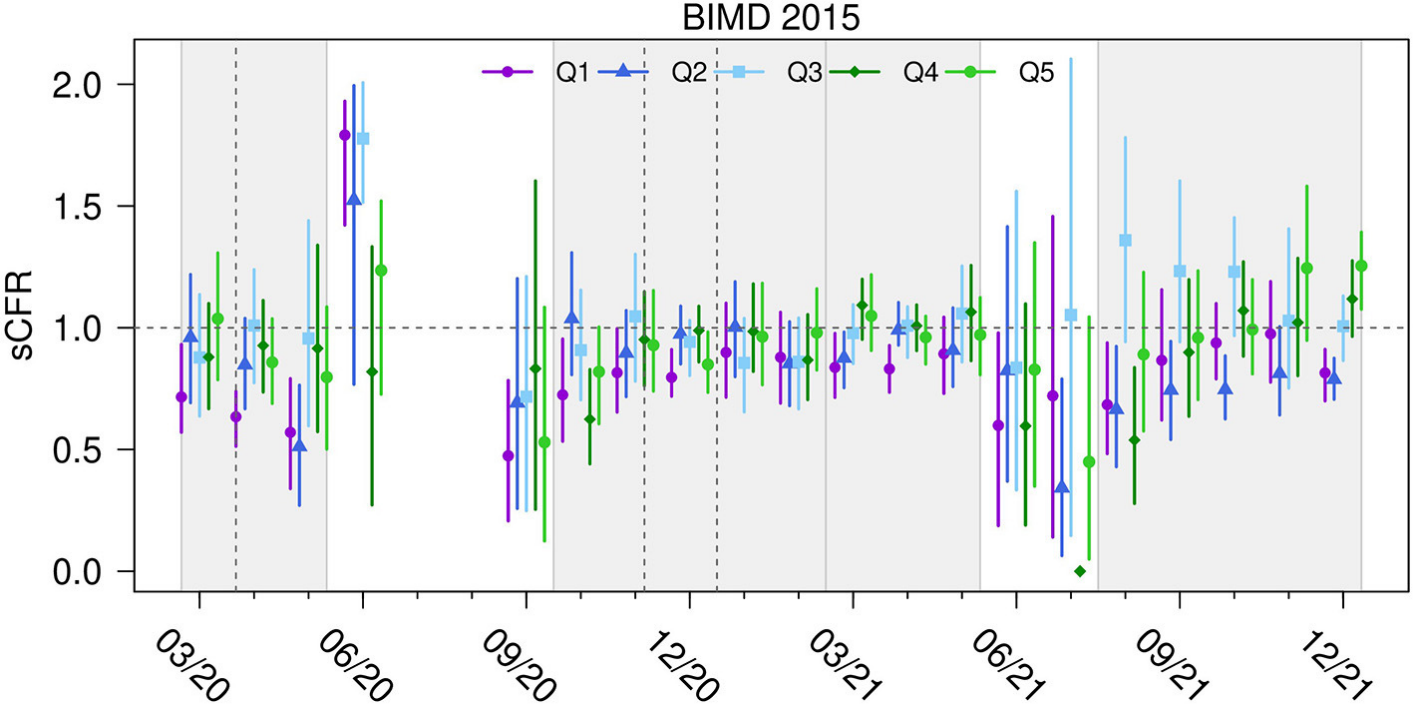
Standardized case fatality ratio for BIMD 2015 quintiles in Bavaria, Germany. Standardized case fatality ratio (sCFR) for COVID-19 for quintiles Q1 (the 20% least deprived districts) to Q5 (the 20% most deprived districts) of Bavarian Index of Multiple Deprivation 2015 (BIMD 2015) between March 2020 and December 2021 in Bavaria, Germany. The time periods of the four infection waves are shown as light gray areas in the figure. The horizontal dashed gray line shows the value of one (“neither increased nor decreased sCFR”). Vertical gray lines show the beginning of the lockdowns.

Figure 5 aggregates the sCFRs monthly across the regions belonging to each BIMD quintile and shows how the area deprivation affects the sCFR at specific time points. There is a general tendency for the least deprived districts to have the lowest sCFR values. The effect is attenuated in the fall and winter of 2020/2021 and seems to be more distinct in the fall and winter of 2021.

## Discussion

This study investigated the association between area deprivation and regional SARS-CoV-2-associated incidence and mortality ratios by districts in Bavaria from the first until the fourth pandemic wave between March 2020 and December 2021. Besides the general view on area deprivation, we also studied the relevance of specific domains on COVID-19-related epidemiological outcomes.

We focused on Bavaria as the largest German federal state by area and the second largest by population size with over 13 million inhabitants, being bigger than many other European countries (for example Sweden, Portugal, the Czech Republic or Greece each have around 10.5 million inhabitants). Bavaria had high infection activity during the COVID-19 pandemic. Infection control measures were uniform in this single federal state, unlike Germany where control measures differ from state to state.

In addition to infection incidences, we examined whether the effects of area-based material and social deprivation were also reflected in mortality. The focus was on standardized ratios (standardized incidence or mortality ratio (SIR/SMR)) taking into account the demographic structure of the regions. We used the Bayesian BYM model, which accounts for the correlation between the two measures. We also used unstructured random effect models for the time-aggregated analysis.

In the unstructured analysis (excluding regional structure and averaging over longer time periods) a positive association was found between the BIMD 2015 and the SIR/SMR. This demonstrates that the COVID-19 burden increases with increasing area deprivation. In relation to the seven area deprivation domains included in the BIMD 2015, income and social capital deprivation were found to be positively associated with incidence and mortality ratios. In addition, a positive association was found between employment deprivation and SMR, while the association between environment deprivation and SIR was negative. These observations are consistent with a corresponding study for Belgium (19).

Our findings are in line with several previous results. The association of area deprivation with the health burden of COVID-19 has been studied in a number of international studies, e. g. in the United Kingdom (10, 47), India (11), Brazil (48), Italy (49), and the United States (12, 13, 50, 51). The study by Bach-Mortensen et al. (10) investigated the association between area deprivation and COVID-19 outbreaks and related deaths among care home residents in England. They found that deaths were more common in the most deprived than in the least deprived areas, while outbreaks in care homes did not vary by area deprivation. Higher social deprivation, quantified using the Townsend Deprivation Score, was found to be associated with greater risk of dying from COVID-19 in another study from the United Kingdom (47). Study results from India and the United States showed that higher SARS-CoV-2 incidences or odds of infection have been found in more deprived compared to less deprived areas (11–13). Studies from Brazil and Italy concerning case-fatality ratio (CFR) and COVID-19 related deaths found a higher CFR and increased risk of death in people living in regions of highest deprivation (48, 49). Comparing rural and urban environments in the United States, a study from Kitchen and colleagues found a positive relationship between area deprivation and COVID-19 prevalence, which was higher for rural counties, when compared to urban ones (50). A combination of area level deprivation data and individual data from two U.S. municipalities was analyzed in a study by Feehan et al. (51). While higher area deprivation was found to be associated with higher risk of SARS-CoV-2 infection, the authors found that individual-level data accounted for a significant proportion of this association.

Interestingly, our results also show that during the first wave higher SIR and SMR were observed in less deprived districts, whereas this association reversed over time. This finding has also been confirmed in German-wide studies (14, 20). The association between infection incidence and social deprivation during the first wave was investigated by Wachtler et al. and Plümper and Neumayer (14, 20). For this purpose, the German Index of Socioeconomic Deprivation from the Robert Koch Institute, Germany's national Public Health institute, was linked to incidence data (14). However, this index considers only three dimensions of deprivation, whereas the BIMD considers seven domains. Across Germany, higher incidences were observed in less deprived regions at the onset of the pandemic, which was associated with affluent ski vacationers returning with SARS-CoV-2 infection. Over time, this effect disappeared in the generally less deprived south of Germany (federal states of Baden-Württemberg and Bavaria), while higher incidences were observed in other more deprived regions. Similar observations were published by Plümper and Neumayer (20), where the authors concluded that COVID-19 started as a rich man's disease and slowly transformed into a poor man's disease.

Socioeconomic differences in infection risk during the second wave were investigated in Germany by Hoebel and colleagues (15). Similar to the first wave, a higher incidence rate was found in less deprived regions at the beginning of the second wave. Again, this pattern reversed as the second wave progressed. In the second wave, COVID-19-related mortality and area deprivation were also examined in Germany, with higher mortality rates found among residents of deprived areas (16). In the third wave of infection, higher incidences were observed in socioeconomically disadvantaged regions (17, 18), which was related to the fact that individuals from socioeconomically disadvantaged regions were not able to limit their mobility as much as individuals from less disadvantaged regions due to their occupation (17). The fourth wave showed a similar association of infections and area deprivation as the second wave, despite the vaccination campaign (18). It is worth mentioning that in our study the overall mortality during the third and fourth waves was reduced compared to the second wave, while no reduction in premature mortality (mortality within persons of an age below 65) was observed. This might be attributable to the vaccination campaign, which was launched on 27/12/2020 in Germany (52). Similar to many other countries, older age groups and the most vulnerable persons were prioritized for the vaccination in Germany. A study by Wollschlaeger et al. (53) in the German federal state of Rhineland-Palatinate found an association between higher vaccination coverage and a decrease in COVID-19 fatalities in the 80+ age group in the 1st months of 2021, supporting our hypothesis. Another study with individual level vaccination data in Bavaria found a high vaccine efficacy in persons over 80 years of age over a similar observation period (54).

In Bavaria, approximately 9% of all COVID-19 attributed deaths were premature deaths, which by definition occur at an age far below the average life expectancy. Unfortunately, the data provided by the LGL did not contain information on personal risk factors or pre-existing medical conditions of the deceased persons. Therefore, the characterization of those who died prematurely remains a task for the future. It should be closely monitored how the number of premature deaths due to/with COVID-19 develops over time, since according to the German health monitoring, premature deaths are avoidable in many cases.

There are several possible reasons for the finding that infection rates increase with increasing area deprivation. People in materially and socially advantaged areas might adhere more to the recommended behavioral changes during the pandemic (55, 56). Such changes include reducing contacts or having better opportunities to work remotely from home, which is still recommended in Germany in spring 2022. As for mortality, the reasons are probably more complex. It is possible that some of the outcomes are related to different prior health burdens (other than COVID-19) in the differently deprived areas. Geographical patterns of health inequalities may also apply to COVID-19 and some unequally distributed chronic diseases may themselves be risk factors for SARS-CoV-2 infection or severe disease course. In Germany, COVID-19 mortality is often assessed only descriptively, and the underlying patterns have not yet been studied in depth. A study from Bavaria investigating the factors related to mortality from COVID-19 in persons over 50 years found SIR to have the greatest effect on the SMR (57).

The district-level BIMD 2015 used in our study was defined for Bavarian districts with varying population sizes (from 40,842 to 1,488,202 inhabitants, median 117,648) and represents a relatively coarse resolution for the deprivation at hand. Individual districts may represent a complex mixture of different settings. However, when using the area-based index, it is assumed that districts are homogeneous within themselves. Therefore, the BIMD 2015 at the district level should be used with caution as a proxy for individual socioeconomic data. A large number of disadvantaged households may account for a few cases, while a few rich households may be responsible for a large number of infections. In terms of aggregated data, the district may have both a high level of deprivation and a high incidence rate, but in fact lower individual socioeconomic deprivation is related to lower incidence. This may carry the risk of ecological bias arising from the assumption that inferences about individual patterns can be drawn from patterns observed in clusters (groups). However, we had to choose the district level since the COVID-19 data in Germany are aggregated only at this spatial level. To our knowledge, there is only one Germany-wide study to date that has investigated differences in the risk of SARS-CoV-2 infection according to socioeconomic position at the individual level (58). Individual socioeconomic position was measured by education and income. The study found that the odds of SARS-CoV-2 infection were significantly increased in adults with low levels of education compared to adults with high levels of education. In terms of income, the odds of infection were higher in low-income individuals than in high-income individuals, although the result was not statistically significant. The data were collected during the second infection wave. These results are consistent with our study on the dimensions of education and income deprivation. Nevertheless, our study shows that there are other additional factors, such as employment and social capital deprivation, that link deprivation to the regional COVID-19 burden.

This is also true for domains such as income and social capital deprivation. However, educational deprivation also shows a westward trend and is identified as an influential aspect of deprivation in our structured analysis of time-specific regional deprivation effects (see Supplementary Figure 8).

In our study, we included population density as a confounding factor, however it did not show much effect on the relationship between area deprivation and SIR/SMR (see Table 1).

Finally, we observed that health inequality (expressed as the ratio of IR or MR between the highest and lowest BIMD quintiles) increased during the 2020 fall and winter periods. Vaccination appears to be associated with a balancing of health inequality between the most and least deprived districts. Lockdown periods, on the other hand, appear to be associated with increased or persistent inequality.

We also examined how an area's deprivation level affects the risk of dying if infected (sCFR). Our data support the hypothesis that in districts with low deprivation, sCFR is also generally lower. During the fall and winter of 2020, the SIR and SMR differ between districts with low and high deprivation, but the risk of dying from infection is less pronounced.

We could not correct for vaccination coverage as another confounding factor in a satisfactory manner and therefore had to refrain from including it in our analyses. Vaccination data only contains information on the place of vaccination but not on the place of residence of the vaccinated persons. As many vaccinations have taken place in city districts, people from the surrounding areas were more likely to travel there to get vaccinated.

Ascertainment bias relates to the underreporting of COVID-19 cases. It is very likely that not all infections were officially reported. Testing strategies were a major focus of the Bavarian government, testing opportunities were easily accessible and evenly distributed across the state. However, these testing strategies have changed over time, from free PCR testing for anyone who wanted to be tested to testing only for health professionals with symptoms in early 2022. It is obvious that the reporting rate is not homogeneous over time. Moreover, continuous serum tests within a representative Bavarian cohort have not been performed. It is also not certain whether the reporting rates estimated in different cohorts at the beginning of the pandemic [as summarized in a meta-analysis (59)] can be transferred to the Bavarian population. When correcting for underreporting, we would assume a constant underreporting rate per month for all of Bavaria. We assume that the variations in the reporting rate would change the relative baseline risk in the BYM model without affecting the relative position of the BIMD 2015 quintiles to each other: The linear trend for the quintiles of BIMD 2015 would remain as shown in the analyses.

Another limitation of this study could be possible confounding of area deprivation and geographical location. It could be that an area bordering Bavaria increases the infection incidence in a Bavarian border district with high deprivation. Border districts may have higher BIMD 2015 values (see Figure 1A). This would artificially strengthen the association between BIMD 2015 and SIR. The BYM model only considers the neighborhood effect within Bavaria and neglects any effects outside the region. Supplementary Figure 3 shows the distribution of the quintiles of deprivation domains across the Bavarian districts. It is easy to see that the BIMD 2015 and some of its domains follow the geographical east and north-east trend. This is also true for domains such as income and social capital deprivation. However, educational deprivation also shows a westward trend and is identified as an influential aspect of deprivation in our structured analysis of time-specific area deprivation effects (see Supplementary Figure 8).

Despite potential neighborhood effects that could come from outside the region of interest, we followed a modeling approach that takes into account the spatial structure of the data (BYM) model. In addition, the bivariate version of the model, as used in the present study, takes into account the correlation between the two endpoints, which is important for the simultaneous modeling of SIR and SMR and makes the analysis of potential effects more powerful. A strong correlation of around 0.7–0.8 between SIR and SMR was found for all models, which suggests that both endpoints have similar geographical risk patterns. It was also found that the unstructured model is able to identify the key drivers of the relationship between area deprivation and incidence and mortality rates, even though no such strong spatial correlation pattern could be specified in the model.

In addition, we explored the different dimensions of deprivation, which allowed us to identify which domains might be affected differently by the pandemic. These findings are consistent with those of other studies, but additional associations between social capital deprivation and, to a lesser extent, environmental deprivation, were detected. It is also interesting to note that deprivation effects became effective when pandemic activity was high: when the crisis intensifies, aspects of social equity become even more important.

Even in a particularly wealthy region of an already economically strong country like Germany, COVID-19 affects residents of differently deprived districts in a different manner. Large differences of up to 41% were found in the SIR and 110% in the SMR between districts in the lowest and highest quintiles, especially in the winter of 2020/2021, suggesting that COVID-19 was a disease that affected disadvantaged areas in the second and third waves. It is also interesting to note that lockdowns have not been able to mitigate the social inequalities associated with deprivation. Therefore, specific strategies need to be explored to successfully control the pandemic in deprived areas.

## Conclusion

This study reports the results of a retrospective ecological study investigating the relationship between area deprivation and SARS-CoV-2-related burden. It quantifies the influence of regional deprivation on SIR and SMR, which were higher in more deprived than in less deprived areas. Higher levels of income, employment, education and social capital were additionally identified as factors reducing COVID-19 disease burden at the district level. Vaccination appeared to balance incidence and mortality rates between the most and least deprived districts. Under lockdown such a compensation was not observed. Vaccination makes an important contribution to health equality.

## Data Availability Statement

The raw data supporting the conclusions of this article will be made available by the authors, without undue reservation.

## Ethics Statement

The studies involving human participants were reviewed and approved by Ethics Committee of the Medical Faculty of the LMU Munich (No. 21-0213 KB). Written informed consent from the participants' legal guardian/next of kin was not required to participate in this study in accordance with the national legislation and the institutional requirements.

## Author Contributions

WM and LS designed the study. KM, UM, and WM acquired the data. WM and UM helped KM with the methodology. KM analyzed the data. KM and WM wrote the first draft of the manuscript. All authors interpreted the data, read, revised, and approved the final manuscript.

## Conflict of Interest

The authors declare that the research was conducted in the absence of any commercial or financial relationships that could be construed as a potential conflict of interest.

## Publisher's Note

All claims expressed in this article are solely those of the authors and do not necessarily represent those of their affiliated organizations, or those of the publisher, the editors and the reviewers. Any product that may be evaluated in this article, or claim that may be made by its manufacturer, is not guaranteed or endorsed by the publisher.
