# Supplementary material for: Area Deprivation and COVID-19 Incidence and Mortality in Bavaria, Germany: A Bayesian Geographical Analysis
Source: Front Public Health. 2022 Jul 15;10:927658. doi: 10.3389/fpubh.2022.927658 (PMC9334899; doi:10.3389/fpubh.2022.927658)
Supplement: Supplementary file 1 [file Data_Sheet_1.docx]

Supplementary Material

# Supplementary Figures and Tables

## Supplementary Figures

**
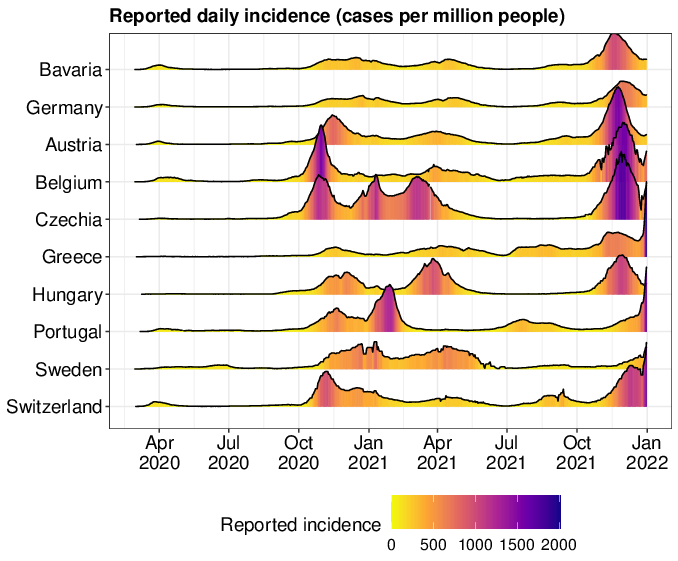
**

**Supplementary Figure 1.** Comparison of daily SARS-CoV-2 incidence in Bavaria compared to Germany and other selected European countries of similar size to Bavaria. The reported daily incidence is presented as a 7-day moving average of the number of cases per million persons between March 2020 and December 2021.


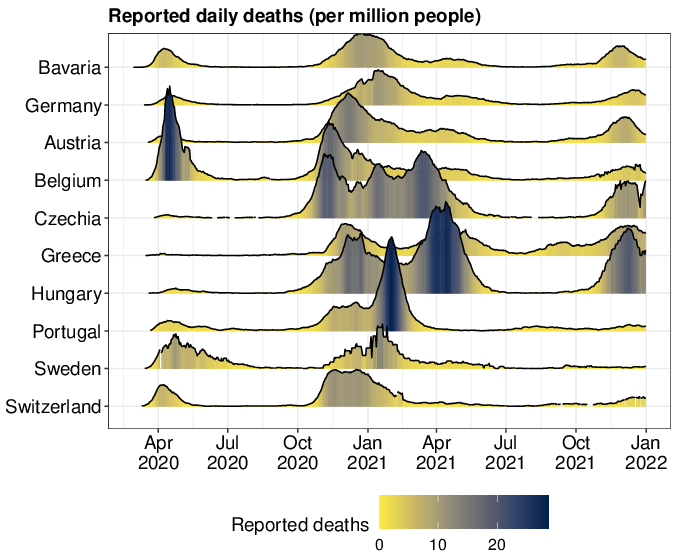


**Supplementary Figure 2.** Comparison of daily SARS-CoV-2 deaths in Bavaria compared to Germany and other selected European countries of similar size to Bavaria. The reported daily deaths are presented as a 7-day moving average of the number of cases per million persons between March 2020 and December 2021.


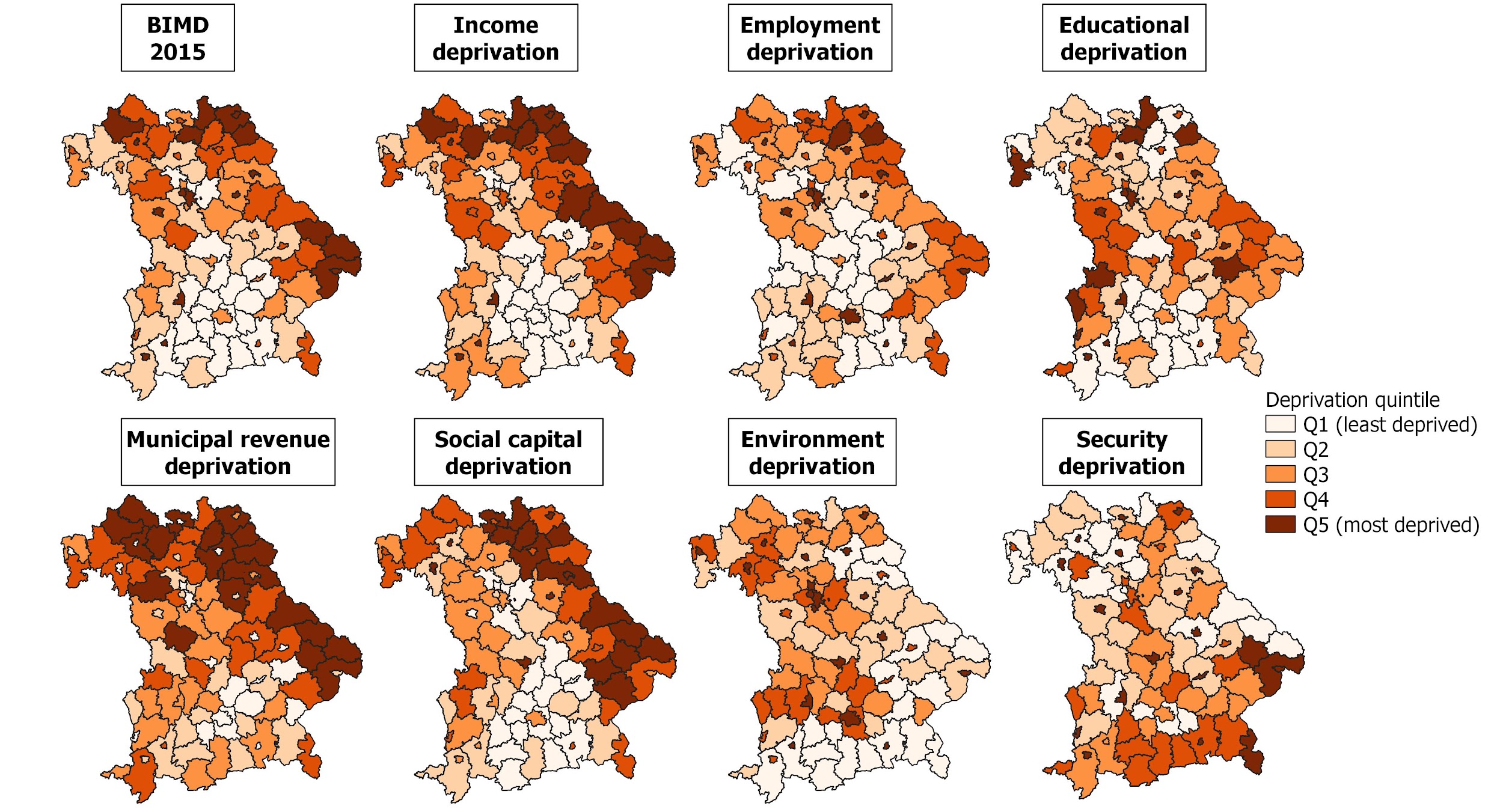


**Supplementary Figure 3.** Bavarian Index of Multiple Deprivation (BIMD) for the reference year 2015 and its domains. The BIMD 2015 is shown together with its domains income, employment, educational, municipal/district revenue, social capital, environment and security deprivation for the 96 districts in Bavaria, Germany. The deprivation quintile Q1 shows the least deprived and the quintile Q5 the most deprived districts.

**
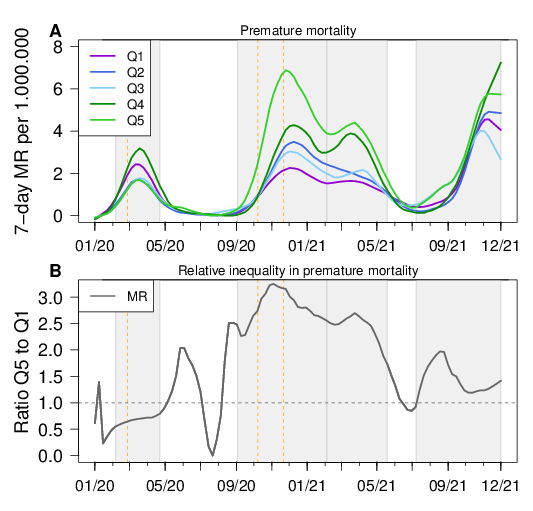
**

**Supplementary Figure 4.** Premature mortality rates for BIMD 2015 quintiles in Bavaria, Germany. (A) Weekly reported premature mortality rates (in SARS-CoV-2 patients aged <65 years) in per 1,000,000 population for districts belonging to each quintile of the Bavarian Index of Multiple Deprivation 2015 (BIMD 2015) between 2015 between January 2020 and December 2021 in Bavaria, Germany. Q1 describes the 20% least deprived and Q5 the 20% most deprived of all 96 districts. (B) The ratio of the curves of Q5 and Q1 shown in (A) over time. The time periods of the four infection waves are shown as light gray areas in the figure. Start dates of lockdowns are indicated as dashed vertical lines. MR = mortality rate.


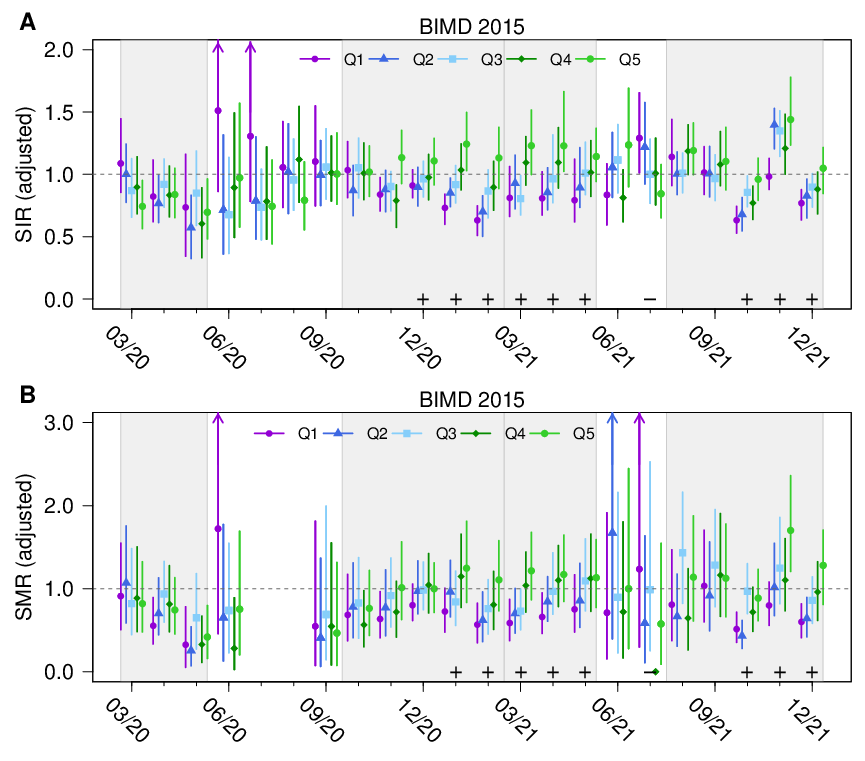


**Supplementary Figure 5.** Standardized incidence and mortality ratios for BIMD 2015 quintiles in Bavaria, Germany. Standardized incidence ratios (SIR, A) and mortality ratios (SMR, B) of SARS-CoV-2 infections and related fatalities for quintiles Q1 (the 20% least deprived districts) to Q5 (the 20% most deprived districts) of Bavarian Index of Multiple Deprivation 2015 (BIMD 2015) between March 2020 and December 2021 in Bavaria, Germany. A plus sign (+) indicates a statistically significant increasing linear trend with increasing deprivation quantile. The time periods of the four infection waves are shown as light gray areas in the figure. The horizontal dashed gray line shows the value of one (“neither increased nor decreased SIR/SMR''). The model is additionally adjusted for population density.

**
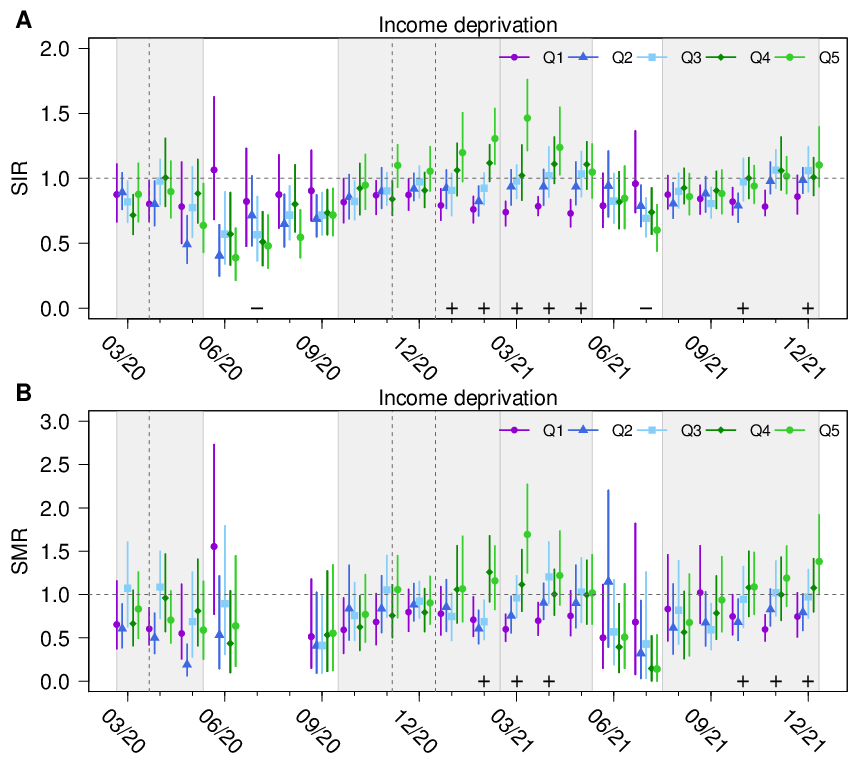
**

**Supplementary Figure 6.** Association of income deprivation and standardized incidence and mortality ratios. Standardized incidence ratios (SIR, a) and mortality ratios (SMR, b) of SARS-CoV-2 infections and related fatalities for the quintiles Q1 (lowest deprivation) to Q5 (highest deprivation) of income deprivation between March 2020 and December 2021 in Bavaria, Germany. A plus sign indicates a statistically significant increasing linear trend with increasing deprivation quantile, a minus sign indicates a statistically significant decreasing linear trend with increasing deprivation quantile. The time periods of the infection waves are shown as light gray areas in the figure. Vertical gray lines indicate the beginning of the lockdowns.


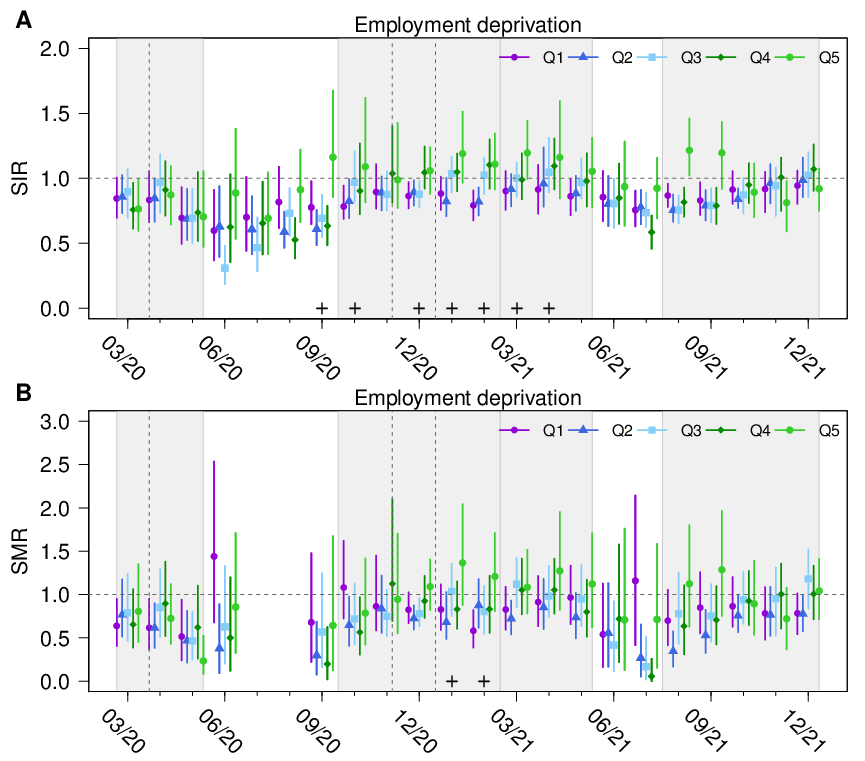


**Supplementary Figure 7.** Association of employment deprivation and standardized incidence and mortality ratios. Standardized incidence ratios (SIR, upper) and mortality ratios (SMR, lower) of SARS-CoV-2 infections and related fatalities for the quintiles Q1 (lowest deprivation) to Q5 (highest deprivation) of employment deprivation between March 2020 and December 2021 in Bavaria, Germany. A plus sign indicates a statistically significant increasing linear trend with increasing deprivation quantile, a minus sign indicates a statistically significant decreasing linear trend with increasing deprivation quantile. The time periods of the infection waves are shown as light gray areas in the figure. Vertical gray lines indicate the beginning of the lockdowns.


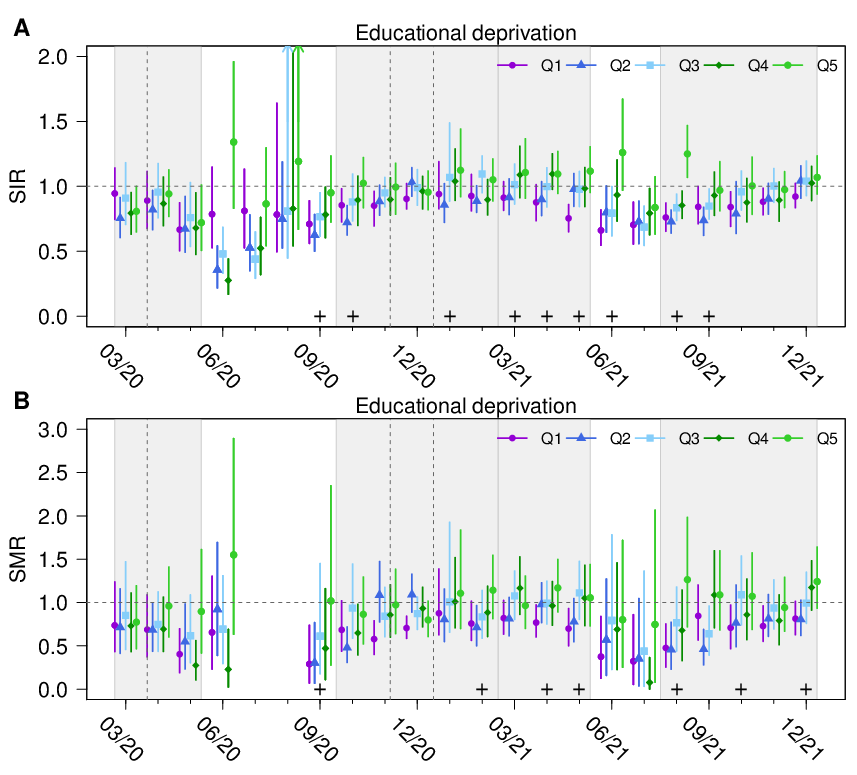


**Supplementary Figure 8.** Association of educational deprivation and standardized incidence and mortality ratios. Standardized incidence ratios (SIR, upper) and mortality ratios (SMR, lower) of SARS-CoV-2 infections and related fatalities for the quintiles Q1 (lowest deprivation) to Q5 (highest deprivation) of educational deprivation between March 2020 and December 2021 in Bavaria, Germany. A plus sign indicates a statistically significant increasing linear trend with increasing deprivation quantile, a minus sign indicates a statistically significant decreasing linear trend with increasing deprivation quantile. The time periods of the infection waves are shown as light gray areas in the figure. Vertical gray lines indicate the beginning of the lockdowns.


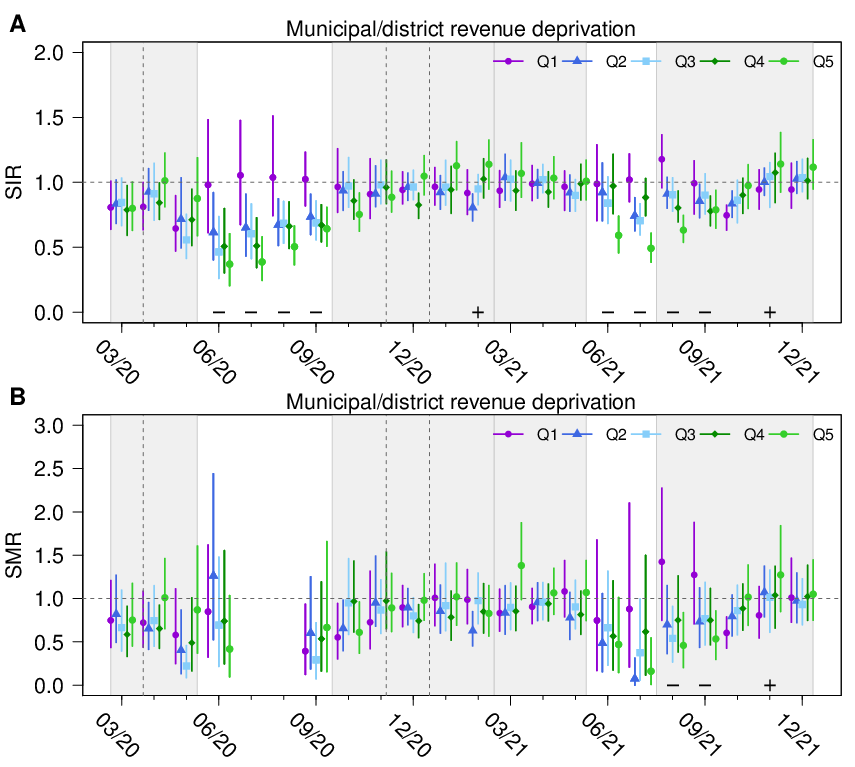


**Supplementary Figure 9.** Association of municipal/district revenue deprivation and standardized incidence and mortality ratios. Standardized incidence ratios (SIR, upper) and mortality ratios (SMR, lower) of SARS-CoV-2 infections and related fatalities for the quintiles Q1 (lowest deprivation) to Q5 (highest deprivation) of municipal/district revenue deprivation between March 2020 and December 2021 in Bavaria, Germany. A plus sign indicates a statistically significant increasing linear trend with increasing deprivation quantile, a minus sign indicates a statistically significant decreasing linear trend with increasing deprivation quantile. The time periods of the infection waves are shown as light gray areas in the figure. Vertical gray lines indicate the beginning of the lockdowns.


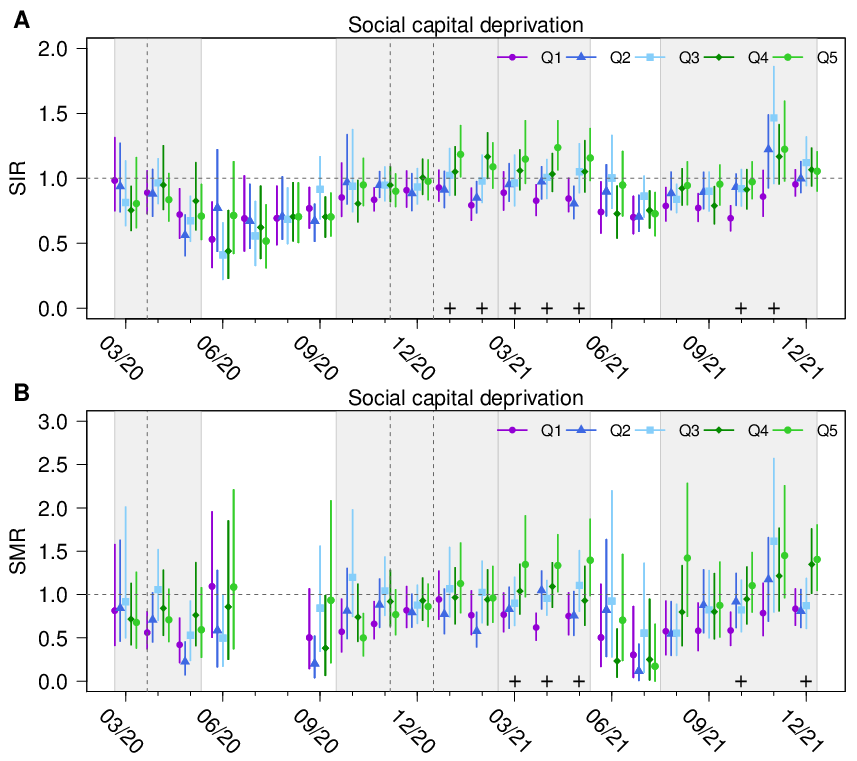


**Supplementary Figure 10.** Association of social capital deprivation and standardized incidence and mortality ratios**.** Standardized incidence ratios (SIR, upper) and mortality ratios (SMR, lower) of SARS-CoV-2 infections and related fatalities for the quintiles Q1 (lowest deprivation) to Q5 (highest deprivation) of social capital deprivation between March 2020 and December 2021 in Bavaria, Germany. A plus sign indicates a statistically significant increasing linear trend with increasing deprivation quantile, a minus sign indicates a statistically significant decreasing linear trend with increasing deprivation quantile. The time periods of the infection waves are shown as light gray areas in the figure. Vertical gray lines indicate the beginning of the lockdowns.

**
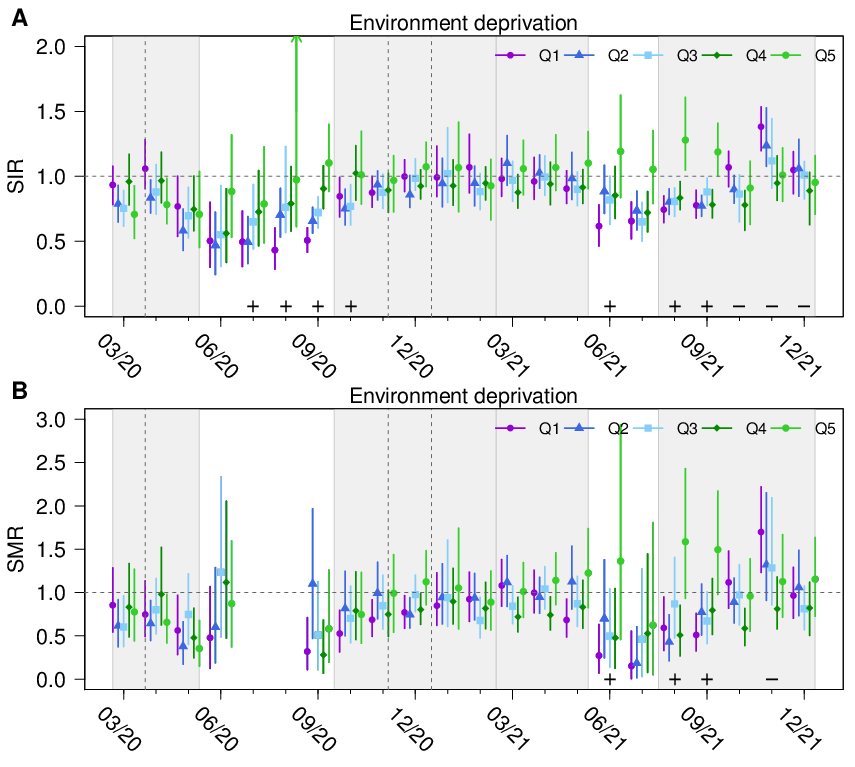
**

**Supplementary Figure 11.** Association of environment deprivation and standardized incidence and mortality ratios**.** Standardized incidence ratios (SIR, upper) and mortality ratios (SMR, lower) of SARS-CoV-2 infections and related fatalities for the quintiles Q1 (lowest deprivation) to Q5 (highest deprivation) of environment deprivation between March 2020 and December 2021 in Bavaria, Germany. A plus sign indicates a statistically significant increasing linear trend with increasing deprivation quantile, a minus sign indicates a statistically significant decreasing linear trend with increasing deprivation quantile. The time periods of the infection waves are shown as light gray areas in the figure. Vertical gray lines indicate the beginning of the lockdowns.


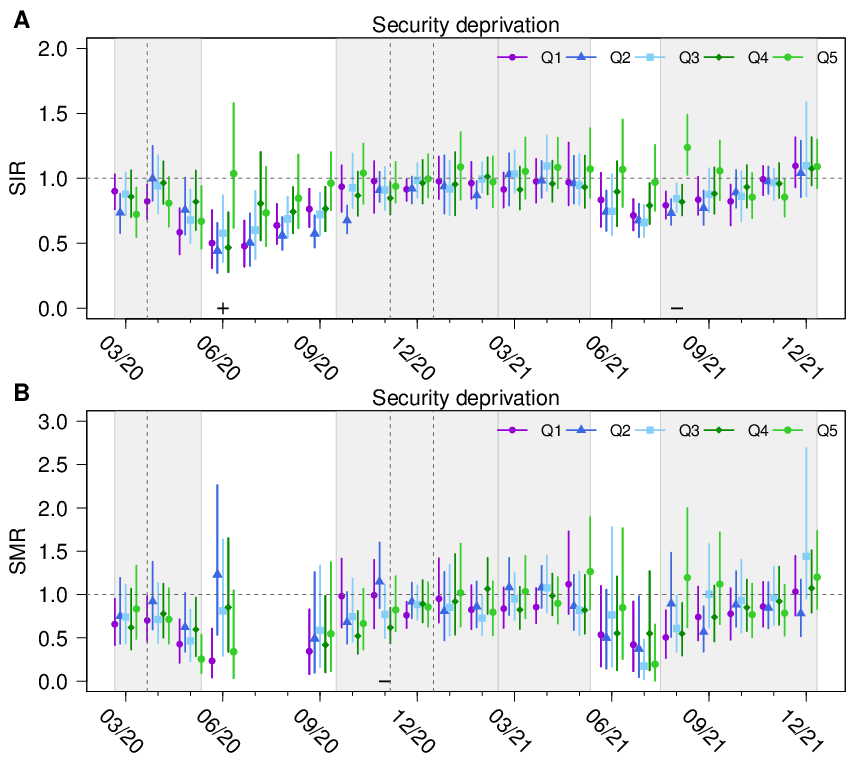


**Supplementary Figure 12.** Association of security deprivation and standardized incidence and mortality ratios. Standardized incidence ratios (SIR, upper) and mortality ratios (SMR, lower) of SARS-CoV-2 infections and related fatalities for the quintiles Q1 (lowest deprivation) to Q5 (highest deprivation) of security deprivation between March 2020 and December 2021 in Bavaria, Germany. A plus sign indicates a statistically significant increasing linear trend with increasing deprivation quantile, a minus sign indicates a statistically significant decreasing linear trend with increasing deprivation quantile. The time periods of the infection waves are shown as light gray areas in the figure. Vertical gray lines indicate the beginning of the lockdowns.

## Supplementary Tables

| **Age group** | **ESP 2013** | **ESP 2013 aggregated** | **Bavaria 2020** |
| --- | --- | --- | --- |
| <1 | 0.01 | 0.01 | 0.010 |
| 1-4 | 0.04 | 0.04 | 0.039 |
| 5-9 | 0.055 | 0.055 | 0.045 |
| 10-14 | 0.055 | 0.055 | 0.044 |
| 15-19 | 0.055 | 0.055 | 0.046 |
| 20-24 | 0.06 | 0.06 | 0.057 |
| 25-29 | 0.06 | 0.06 | 0.064 |
| 30-34 | 0.065 | 0.065 | 0.069 |
| 35-39 | 0.07 | 0.07 | 0.065 |
| 40-44 | 0.07 | 0.07 | 0.062 |
| 45-49 | 0.07 | 0.07 | 0.063 |
| 50-54 | 0.07 | 0.07 | 0.079 |
| 55-59 | 0.065 | 0.065 | 0.081 |
| 60-64 | 0.06 | 0.06 | 0.068 |
| 65-69 | 0.055 | 0.105 | 0.102 |
| 70-74 | 0.05 |  |  |
| 75-79 | 0.04 | 0.09 | 0.105 |
| 80-84 | 0.025 |  |  |
| 85+ | 0.025 |  |  |
| **Total** | **1** | **1** | **1** |

**Supplementary Table 1.** Comparison of the European Standard Population 2013 (ESP 2013) and the Bavarian population of the year 2020. The weights are shown for each age group. The column ESP aggregated shows the weight in the same age groups as the Bavarian population 2020.
